# Lead Me Not into Temptation: Using Cognitive Reappraisal to Reduce Goal Inconsistent Behavior

**DOI:** 10.1371/journal.pone.0039493

**Published:** 2012-07-24

**Authors:** Véronique Leroy, Jacques Grégoire, Eran Magen, James J. Gross, Moïra Mikolajczak

**Affiliations:** 1 Department of Psychology, Université Catholique de Louvain, Louvain-la-Neuve, Belgium; 2 Counseling & Psychological Services, University of Pennsylvania, Philadelphia, Pennsylvania, United States of America; 3 Department of Psychology, Stanford University, Stanford, California, United States of America; 4 Department of Psychology, Université Catholique de Louvain, Louvain-la-Neuve, Belgium; Hungarian Academy of Sciences, Hungary

## Abstract

Temptations besiege us, and we must resist their appeal if we are to achieve our long-term goals. In two studies, we tested the hypothesis that cognitive reappraisal could be used to successfully maintain performance in a task embedded in temptation. In Study 1, 62 participants had to search for information on the Internet while resisting attractive task-irrelevant content on preselected sites. In Study 2, 58 participants had to count target words in a funny TV sequence. Compared to the no-reappraisal condition, participants who understood the situation as a test of willpower (the reappraisal condition) (1) performed better at the task (Studies 1 and 2), and (2) were less tempted by the attractive content of the TV sequence (Study 2). These findings suggest that, by making the temptation less attractive and the task more appealing, cognitive reappraisal can help us resist temptation.

## Introduction

*“Mastering others is strength. Mastering yourself is true power”.*

*Lao Tzu*

Whatever our goals, our life satisfaction heavily depends upon our making progress towards these goals [1]–[2]. One obstacle to such progress is temptation, which can be defined as the impulse to behave in a way that will later be regretted [3]. Temptation prioritizes a secondary goal (e.g., relaxing by watching television) over a more significant goal that is deferred in time (e.g., studying to pass an examination). The failure to resist temptation has deleterious consequences in all major life domains [4], including psychological well-being, social adjustment, work performance, and even physical health.

Given the many problems associated with succumbing to temptation, researchers have made great efforts to understand why we indulge temptation and how we can resist it [5]. Classic work by Mischel and his colleagues suggests that temptation can be overridden through attention control [6]: withholding attention (either physically or mentally) from the immediate reward decreases its salience, thereby pre-empting the temptation. Unfortunately, this strategy consumes cognitive and self-regulatory resources, leaving the individual with fewer resources for other cognitive tasks or self-regulatory endeavors [7].

More recently, Magen and Gross [3] proposed an alternate strategy to harness temptation: cognitive reappraisal, or changing the meaning of the situation so as to decrease the value of the temptation and/or increase the value of the task. In a representative study that tested the value of cognitive reappraisal, participants had to take a math test while being tempted by entertaining comedy clips on TV. Half of the participants were instructed to cognitively reappraise the situation as a “test of willpower”. Such reappraisal aimed to decrease the value of the temptation (“If I indulge, it means I don't have willpower”) and increase the value of the task (“If I concentrate, it means I have willpower”). The reappraisal creates a new immediate and desirable goal (proving I have willpower) which competes with the immediate undesirable goal produced by temptation [3]. Reappraisal therefore alters the contingency between the goals with which the individual is faced.

Findings indicated that, compared to the no-reappraisal group, participants in the reappraisal group (1) spent less time watching the comedy clips, and (2) experienced less pleasure when they were watching the comedy clips. This study demonstrated that cognitive reappraisal can be an effective strategy for resisting temptation. However, there is one major limitation of this prior study. It is not clear whether this cognitively-based strategy has any deleterious impact on a concurrent task. If cognitive reappraisal allows individuals to resist temptation, but at the expense of the task's completion, using it as a strategy is counterproductive. Reappraisal can only be considered as an efficient strategy for managing temptation if performance levels can be maintained in completing the task.

In order to test whether using cognitive reappraisal to resist temptation would impact negatively on a concurrent task, we conducted two studies. To ensure the temptations could not be ignored we recreated two laboratory situations in which temptations were embedded in the task. The first study measured the impact of cognitive reappraisal on participants' performance while they were researching information on the Internet. The second study measured the impact of cognitive reappraisal on participants' performance and susceptibility to temptation by having them count target words in an attractive TV sequence.

## Experiment 1

The goal of the present study was to examine the impact of reappraisal on performance (1) in an ecologically valid context (searching for information on the Internet, without being distracted by unrelated tempting content, is a task frequently encountered by students and workers), and (2) where temptation is inherent to the task (both goal consistent and goal inconsistent material are presented on the same page), so that temptation cannot be ignored. We hypothesized that, compared to participants in a control condition, participants who reappraised the task as diagnostic of an internal quality (willpower) would perform better in the task. In order to demonstrate the strength of the temptation in this situation, a pilot study was conducted.

### Methods

#### Participants

Sixty-two undergraduate psychology students (52 women) participated in Study 1 in exchange for course credit (*mean age*  = 19.75 years old; *SD*  = 2.34 years). All participants were over 18 years old. The experiment was conducted in accordance with the Declaration of Helsinki and was approved by the “Psychological Department Ethical Committee” of the Université catholique de Louvain, Belgium. The researcher signs a charter by which s/he commits to informing the participants of their rights (e.g., stopping the experiment without justification). The ethics committee approved the use of oral consent in this study.

#### Procedure

Participants were tested individually in the laboratory. They were told that the study was concerned with the influence of web design and web ergonomics on consumer behavior. They were invited to sit in front of a computer and were presented with a page listing 50 websites with attractive content (i.e., gossip about movie stars, movie theater programs, fashion trends, etc.). They were asked to visit one website after another (in the order provided on the list).

The first task (Task 1) provided an independent measure of the participants' ability to perform the Internet task in the presence of temptation (i.e., websites were preselected on their degree of their content's attractiveness). Participants had 12 minutes to visit as many websites as they could and then answer five questions about each of them (measures of performance).

Prior to the second task (Task 2), participants were randomly assigned to either the experimental condition (reappraisal: n = 31) or the no-reappraisal condition (n = 31). In the reappraisal condition, participants received the following instructions: “*You have done half of the study. I will now ask you to perform the same task but for a new website list. This next task aims to assess your willpower. Please do your best to answer as many questions as possible. Once again, you have 12* *minutes.”* In the no-reappraisal condition, they were told: *“You have done half of the study. I will now ask you to perform the same task but for a new website list. Please do your best to answer as many questions as possible. Once again, you have 12* *minutes.”* Participants then had 12 minutes to visit as many new websites as possible and answer five questions about each. Upon completion of Task 2, participants completed a questionnaire assessing their use of reappraisal.

#### Materials

In order to confirm that the selected websites were tempting, 29 additional undergraduate students were recruited to evaluate the presence of temptation (“I'm tempted to click on the links on this site”, “I'm tempted to read the contents of this site”) and the level of resistance required to complete their task (“I would have preferred to read the contents of the site rather than answer the five questions related to the design and ergonomics of this website”, “I would have preferred to click the links on this site rather than answer the five questions related to the design and ergonomics of this website”). Findings confirmed that the preselected websites can be considered as a temptation, F(1, 57)  = 28.957; p<.001, and that our task implies active resistance to temptation, F(1, 57)  = 44.410; p<.001.

### Measures

#### Manipulation check variable: Task reappraisal

Participants rated their agreement with the statement “*During the task, I thought of the situation as a test of willpower”* on a 7-point scale ranging from 1 (not at all) to 7 (very much). In order to ensure that this question did not act as a reappraisal manipulation, it was asked about each of the two tasks retrospectively (i.e., at the end of the whole experiment, not at the end of each individual task).

#### Task performance

Five questions related to the design and ergonomics of the website (e.g., *Is there a search engine on the website?*) were asked about each of the 50 websites. An overall score was calculated (1 point for each correct answer).

### Results and Discussion

#### Manipulation Checks

Independent samples t-tests showed that the groups' performance did not differ significantly on Task 1 (pre-manipulation task). To test whether our manipulation had the intended effect on task construal, we ran repeated-measures ANOVAs on task reappraisal. As predicted, analyses showed a significant Time x Condition interaction, *F*(1, 60)  = 21.51, *p*<.001, *η^2^_p_* = .26. While the groups appraised the situation similarly on Task 1, *F*(1, 61)  = .01, *ns*, they differed after the manipulation; the reappraisal group (*M* = 5.06; *SD*  = 1.69) viewed the situation as a test of willpower significantly more than the no-reappraisal group (*M* = 3.13; *SD* = 2.43), *F*(1, 61)  = 13.23, *p*<.01, *d* = −.32.

#### Does Reappraisal Influence Task Performance ?

**Figure 1:**
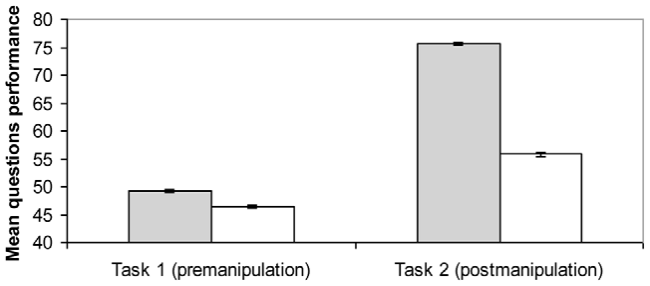
Mean and standard errors of the scores on performance, before and after manipulation by group (Study 1).

A repeated-measures ANOVA, with Time (pre-manipulation vs. post-manipulation) as a within-subject factor and Condition (reappraisal vs. no-reappraisal) as a between-subject factor was conducted to test the effect of reappraisal on performance. We first observed a significant effect of time, *F*(1, 60)  = 223.22, *p*<.001, *η^2^_p_* = .79, showing that performance globally increased over time, and a significant effect of condition, *F*(1, 60)  = 16.46, *p*<.001, *η^2^_p_* = .22, indicating that the reappraisal group (*M* = 75.58; *SD*  = 10.86) performed better than the no-reappraisal group (*M* = 55.77; *SD*  = 15.14). Crucially, we also observed a Time x Condition interaction, *F*(1, 60)  = 50.31, *p*<.001, *η^2^_p_* = .46. To determine the source of this interaction, we compared performance changes across groups (Performance on Task 2 minus Performance on Task 1). As expected, the changes were significantly greater in the reappraisal group (*M* = 26.35; *SD*  = 9.76) than in the no-reappraisal group (*M* = 9.39; *SD*  = 9.07), *t*(60)  = −6.84, *p*<.001, *d* = −1.20. Thus, cognitive reappraisal was effective in increasing performance (See Figure 1).

#### Summary

Compared to the control group, participants who reappraised the task to research information as a test measuring their willpower performed better. This indicates that cognitive reappraisal is effective and does not entail a negative impact on performance. Although an independent sample of participants confirmed that these websites were tempting, the present study did not provide a direct measure of participants' susceptibility to temptation. Study 2 was designed to address this gap.

## Experiment 2

The main goal of Study 2 was to replicate results of Study 1 and extend them in three ways (1) obtaining a baseline estimate of temptation; (2) using a temptation embedded in the task, with clearly distinct measures of temptation and task performance; (3) measuring the self-control cost of using cognitive reappraisal.

Compared with the control condition, we hypothesized that cognitive reappraisal (1) would lead to better performance, (2) would lead to less susceptibility to temptation, and (3) would not significantly deplete self-control resources.

### Methods

#### Participants

Fifty-eight undergraduate psychology students (50 women) participated in this experiment in exchange for course credit (*mean age*  = 19.09 years old; *SD*  = 1.37 years).

#### Procedure

Participants were tested individually in the laboratory. They were told that the study was about “the effect of the word order in a sentence on word recognition”. They were invited to sit in front of a computer and perform a practice trial. The practice sequence was composed of two funny advertisements and a comedy clip (5 minutes), preselected on their degree of their content's attractiveness. They were asked to press a button each time they heard the word “Mum” during the sequence. This task provided us with an independent measure of the participants' selective attention in the presence of temptation.

Prior to the main task, participants were randomly assigned to either the experimental condition (reappraisal: n = 29) or the no-reappraisal condition (n = 29). In the reappraisal condition, participants received the following instructions: “*You have done half of the study. I will now ask you to perform the same task but for a new sequence and with a new target word: “Already”. This next task aims to assess your willpower. Please do your best to hear each target word.”* In the no-reappraisal condition, they were told: *“You have done half of the study. I will now ask you to perform the same task but for a new sequence and with a new target word: “Already”. Please do your best to hear each target word.”* The main sequence was composed of three funny advertisements and two comedy clips (7 minutes 30 seconds). Afterwards, participants completed a measure assessing their susceptibility to temptation and a questionnaire assessing the perception of temptation in the two sequences (training and main sequence) and the use of reappraisal. Afterwards, participants were thanked for their participation and smarties were proposed (Measure of ego-depletion).

### Measures

#### Manipulation check variables

(1) *Selective attention*. The number of time participants heard the target word “Mum” (press on the button) during the first sequence was the measure of selective attention. An overall score was calculated (1 point for each correct answer – Maximum: 17); (2) *Temptation perception*. In order to ensure that the sequences constituted a temptation, participants rated the degree to which the content of the two sequences was tempting on a 10-point scale ranging from 1 (not at all) to 10 (very much); (3) *Task reappraisal*. Participants rated their agreement with the statement “*During the task, I thought of the situation as a test of willpower”* on a 7-point scale ranging from 1 (not at all) to 7 (very much). In order to ensure that this question did not act as a reappraisal manipulation, it was asked about each of the two tasks retrospectively (i.e., at the end of the whole experiment).

#### Outcome variables

(1) *Task performance*. The number of times participants heard the target word “already” (press on the button) was the measure of performance. An overall score was calculated (1 point for each correct answer; maximum  = 16); (2) *Susceptibility to temptation*. Participants answered 25 questions about the content of the main sequence (Example: What was the name of the main character?; 1 point for each detail recalled; maximum  = 25 points); (3) *Ego-depletion*. Amount of smarties taken by the participant (in grams).

### Results and Discussion

#### Manipulation Checks

In order to ensure that the sequences can be considered as a temptation, we compared responses on the item measuring temptation perception of practice (*M* = 7.69; *SD*  = 1.14) and main sequence (*M* = 7.62; *SD*  = .96) to the midpoint of the 10-point Likert scale (5). Participants, on average, responded significantly higher than the middle (neutral) point of the scale (Training: *F*(1, 115)  = 321.40; *p*<.0001; Main sequence: *F*(1, 115)  = 433.96; *p*<.0001), indicating that both practice and main sequences were found tempting.

Independent samples t-tests showed that the experimental conditions did not differ significantly in the practice sequence, either in selective attention, *F*(1, 48)  = .061; *ns* or in temptation perception, *F*(1, 57)  = .052; *ns*. To test whether our manipulation had the intended effect on task construal, we ran repeated-measures ANOVAs on task reappraisal. As predicted, the analyses showed a significant Time x Condition interaction, *F*(1, 56)  = 110.85, *p*<.0001, *η^2^_p_* = .66. While groups appraised the situation similarly during the practice, *F*(1, 57)  = .305, *ns*, they differed after the manipulation: the reappraisal condition (*M* = 5.17; *SD*  = 1.56) viewed the situation as a test of willpower significantly more than the no-reappraisal condition (*M* = 1.86; *SD*  = 1.62), *F*(1, 57)  = 62.85; *p*<.001, *d* = .60.

#### Does Reappraisal Influence Task Performance?

**Figure 2:**
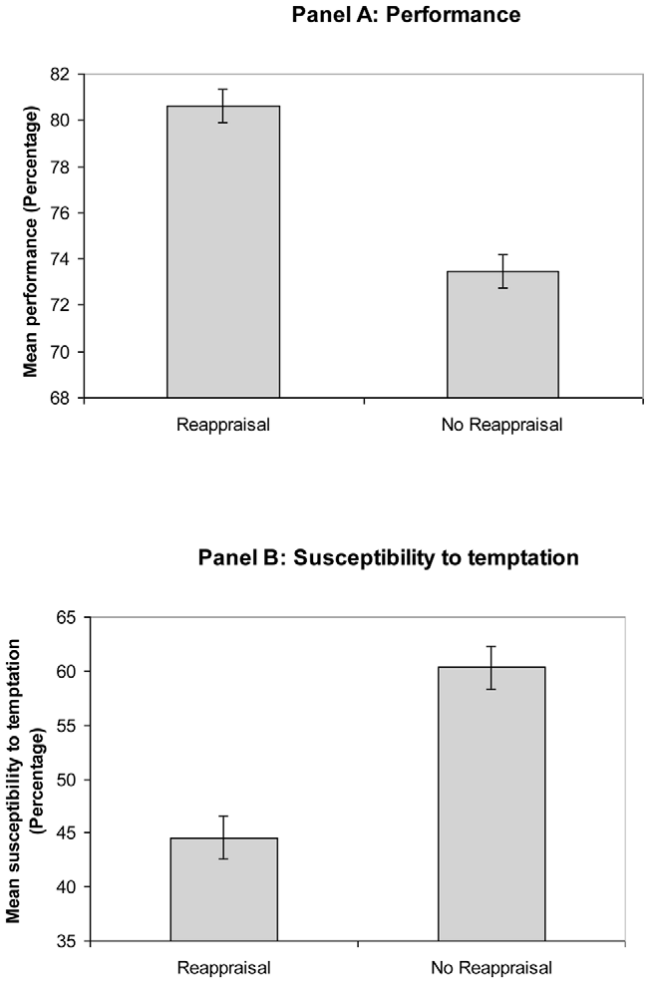
Mean and standard errors of the scores on performance (Panel A) and susceptibility to temptation (Panel B) by group (Study 2).

ANOVA showed that participants in the Reappraisal condition (*M* = 12.90, *SD*  = 1.45) heard more target words during the main sequence than those in the Control condition (*M* = 11.76, *SD*  = 2.20), *F*(1, 57)  = 5.42, *p*<.05, *d* = .20, indicating that performance was higher in the Reappraisal condition than in the Control condition. The cognitive reappraisal manipulation was thus effective in raising performance (see Figure 2 Panel A).

#### Does Reappraisal Influence Temptation?

ANOVA showed that participants in the Control condition (*M* = 15.69, *SD*  = 4.09) recalled more information about the main sequence than those in the Reappraisal condition (*M* = 11.59, *SD*  = 4.40), *F*(1, 57)  = 13.52, *p*<.001, *d* = .52, suggesting that participants in the Control condition spent more time listening to the content of the main sequence. The cognitive reappraisal manipulation was thus effective in reducing temptation (see Figure 2 Panel B).

#### Does Reappraisal Deplete Self-control Resources?

If using cognitive reappraisal depletes self-control resources, participants in the Reappraisal condition (*M* = 1.79, *SD*  = 1.08) should take more smarties (to refill resources) than participants in the Control condition (*M* = 2.45, *SD*  = 2.47). ANOVA showed that the two conditions did not differ significantly on the amount of smarties taken at the end of the experiment, *F*(1, 57)  = 1.709, *ns*. So, participants who reappraised the situation and resisted to temptation did not consume more self-control resources than participants of the control condition who gave in to temptation.

#### Summary

Compared to the control group, participants who reappraised the situation as an opportunity to measure their willpower (1) performed better and (2) succumbed less to the temptation embedded in the contents of the TV sequence. These findings replicate and specify the results provided by Study 1.

## Discussion

Temptation is one of the largest obstacles to the pursuit of goals in everyday life. In the studies described, participants were asked to perform a task while being faced with temptations hard to ignore: the attractive content of websites (Study 1) or of a TV sequence (Study 2). The originality of the two studies lies in assessing task performance. To ensure temptations could not be ignored, we used temptations embedded in a task. The results show that participants who used reappraisal performed better (Studies 1 and 2) and were less distracted by the TV content than the participants of the control group (Study 2).

The fact that reappraisal not only decreased temptation but also increased performance confirms that this strategy is efficient and does not bear significant cognitive costs [8]. Cognitive costs would imply that the reduction in temptation came at the expense of a decrease in performance; but this was not the case. Thus, self-regulation through cognitive reappraisal seems to be not only behaviorally beneficial (a decrease in temptation), but also cognitively beneficial (an increase in performance).

This may explain why Leroy and Grégoire [9] recently found that the recurring use of reappraisal by university students was positively correlated to the average grade point. As suggested by Magen and Gross [3], reappraisal seems to inoculate participants against the potentially deleterious effects of self-control. This strategy could therefore be considered as complementary to attention control [10], by replacing it when contact with temptation is unavoidable.

While this research shows that reappraisal is a promising strategy for harnessing temptation, future studies will need to extend the findings. First, our sample was predominantly composed of female students. Future work would benefit from replicating these results with a larger and more heterogeneous sample. This would also allow to test if the effectiveness of reappraisal depends on individual characteristics (e.g., the ability to change one's initial appraisal of a situation). As pointed out by an anonymous reviewer, the efficiency of reappraisal may also depend on its relevance to the participant's values. In the context of medical research on pain tolerance for example, it may be observed that some participants might tolerate pain longer when it is framed as test of their “willpower”, while others might show increased persistence when their performance is presented as instrumental in “increasing our ability to alleviate the pain of cancer patients.” In short, it may well be that there are not only “main effects” across different forms of reappraisal/reframing, but also interactions with the personal values of participants.

A second important research direction concerns the duration of the effects: how long do the effects of a particular reappraisal episode last? Examining the duration of the effects is particularly important to determine how often people will need to “refresh” the reappraisal to resist a particular temptation.

Third, our results confirm that reappraisal is effective, even when participants cannot ignore the temptation. One explanation for why participants who reappraised were quicker at the task is that they were less distracted (that is to say less tempted) than those who did not reappraise the situation; however, future studies will be necessary to fully document the role of distraction (and other) possible mechanisms.

Finally, our study examined a single form of reappraisal (willpower). It would be interesting to see if other forms of reappraisal (e.g., a predictive test of academic success or a measure of maturity) produce the same effects as those observed in the present study.

This line of research appears particularly promising as we must continually resist the appeal of temptations if we are to achieve our long-term goals. By making the temptation less attractive and the task more appealing, cognitive reappraisal seems to represent a powerful aid to goal striving. Educating people in cognitive reappraisal could help them to harness temptation in a multitude of domains of life such as health promotion (e.g., prevention of smoking) or education (e.g., prevention of procrastination).
